# Multifractal Spatial Patterns and Diversity in an Ecological Succession

**DOI:** 10.1371/journal.pone.0034096

**Published:** 2012-03-21

**Authors:** Leonardo Ariel Saravia, Adonis Giorgi, Fernando Momo

**Affiliations:** 1 Instituto de Ciencias, Universidad Nacional de General Sarmiento, Los Polvorines, Buenos Aires, Argentina; 2 CONICET, Departamento de Ciencias Básicas, Universidad Nacional de Luján, Luján, Buenos Aires, Argentina; Universitat Pompeu Fabra, Spain

## Abstract

We analyzed the relationship between biodiversity and spatial biomass heterogeneity along an ecological succession developed in the laboratory. Periphyton (attached microalgae) biomass spatial patterns at several successional stages were obtained using digital image analysis and at the same time we estimated the species composition and abundance. We show that the spatial pattern was self-similar and as the community developed in an homogeneous environment the pattern is self-organized. To characterize it we estimated the multifractal spectrum of generalized dimensions *D_q_*. Using *D_q_* we analyze the existence of cycles of heterogeneity during succession and the use of the information dimension *D_1_* as an index of successional stage. We did not find cycles but the values of *D_1_* showed an increasing trend as the succession developed and the biomass was higher. *D_1_* was also negatively correlated with Shannon's diversity. Several studies have found this relationship in different ecosystems but here we prove that the community self-organizes and generates its own spatial heterogeneity influencing diversity. If this is confirmed with more experimental and theoretical evidence *D_1_* could be used as an index, easily calculated from remote sensing data, to detect high or low diversity areas.

## Introduction

We know that space can be an essential factor controlling species' coexistence and biodiversity in many communities [1]. Spatial pattern can be the result of environmental heterogeneity or can be produced by species' internal dynamics and interactions [2]. The latter are called self-organized and could be an important determinant of the structure and functioning of ecosystems [3]. Understanding the self-organization of ecological communities and the levels of biological diversity that emerge represents a fundamental theoretical challenge for ecologists [4].

Most studies focus on the effects of spatial patterns or spatial structure on diversity [5], [6] but can we know something about biodiversity by studying spatial pattern?

Biodiversity is usually related to environmental spatial heterogeneity or habitat complexity [7], [8] but self-organizing ecosystems generate its own spatial patchiness. This can be seen in the distribution of canopy height in tropical forests [9]. These distributions are fractals and are suspected to be produced by self-organization [10]. The auto-generated spatial heterogeneity could in principle have a strong influence on diversity, but this was never investigated. If the self-organizing process acts over an ecological succession it may influence both diversity and spatial pattern.

Early studies suggested that a measure of spatial pattern could be used as an index of persistence of populations and successional stage [11], [12]. This measure was the fractal dimension of patches: early successional stages should have a higher fractal dimension because they have more intricate shapes and they are less persistent; late successional stages should have a lower fractal dimension because they have more regular shape and they are more persistent. This relationship between shape and persistence is derived from modified Brownian diffusion models, but it could be applied without committing to any particular Brownian model. Though its application was promising [13] no further studies of fractal dimension followed this line (but see [14]).

Spatial pattern and succession has been linked by a conceptual model called nucleation [15], where some species act as a nucleus to facilitate the establishment of other species; patches of the colonizing species first grow and then start to decrease as they are replaced by other, late successional, species. Eventually patches of some species coalesce and the spatial pattern is relatively fixed unless some disturbance occurs. This was later developed as a “model of heterogeneity cycles” [16] where cycles of heterogeneity alternate through succession: high heterogeneity represents periods of species invasion and establishment, and low heterogeneity represents periods of exclusion. One problem with this approach is that the measurement of heterogeneity is scale dependent so different sampling units will give different results [17].

Fractals methods can provide indices that measure the spatial heterogeneity of the community independent of the scale of observation over a range of scales [18], [19], and have been extensively used in ecology [20], [21]. A natural extension of these is multifractal analysis [22] that has not been applied so widely to date. Fractal analysis usually looks at the geometry of sets, that is, patterns arising from presence/absence data, whereas multifractal analysis looks at the arrangement of quantities, like densities or proportions.

In ecology, multifractals have been used in the analysis of temporal variability in plankton biomass [23], [24], as a model of extinction and the origin of species [25], in the analysis of the spatial distribution of gaps caused by falling trees in the rainforest [26], [27], and more recently in the analysis of species-area relationships [28], [29]. The biomass spatial distribution of intertidal microphytobenthos has been analyzed using a modified multifractal technique [30]. These communities can be used as a model to study the relationship between species diversity, succession and spatial pattern. In a broader sense these communities are called periphyton.

Periphyton grows attached to submerged surfaces and is a complex community composed of microorganisms and other components including algae, bacteria, fungi, animals, and inorganic and organic detritus [31]. It can be responsible for most primary production in shallow aquatic environments such as streams, lakes, coastal waters and wetlands [32], [33].

One of the descriptive characteristics of periphyton communities is biomass, which is defined as the amount of organic matter (or carbon) that has accrued from the production per unit area [34]. Biomass is a measure that integrates the interactions of individual characteristics of species, abiotic environmental controls and effects of herbivory [35].

In turn, the spatial distribution of biomass is the result of colonization processes, growth, competition and herbivory, to mention only the most important ones. Colonization occurs in random places that act as nuclei for development of algal biomass. As growth progresses algal communities develop a complex architecture similar to that of tropical forests, where competition for light and nutrients plays an important role [36].

Thus we use experimental microcosms with attached microalgae communities (periphyton) to explore the relationship between spatial pattern and diversity throughout succession. Our hypothesis is that spatial self-organizing processes, characterized by multifractals, influence diversity over ecological succession. We also discuss the application of the conceptual model of cycles of heterogeneity during succession.

## Methods

### Experimental procedures

The periphyton colonization was carried out on squares of 5×5×0.1 cm high impact polystyrene. Initially we added 20 squares at the bottom of one 60×60 cm glass aquarium and then 10 squares were added every week for six weeks. The experimental device was located in a controlled environment with a light period of 12 hours and a temperature of 20°C. The aquarium was filled with water filtered through a 30 µM pore mesh. After that we added 50 cm3 of water with high density of algae obtained by scraping *Egeria densa* plants to remove the natural periphyton community. Twice a week we added water with periphytic algae to compensate for evaporation, to accelerate colonization and to simulate natural conditions. In the seventh week, sixty artificial substrata were removed and photographed to estimate species' composition and abundance. In one square for each week a Nikon Optiphot microscope with an underwater lens and phase contrast was used to identify algae. A minimum of ten random fields were observed, and observation continued on subsequent fields until no new species was found. Algal density was then calculated, together with species richness and Shannon diversity index [37].

To obtain the biomass spatial pattern, the photographs were digitized and the brightness values of each pixel were converted to biomass following the method of Saravia et al. [38]. Thus we obtained the spatial distribution of the periphyton biomass, once a week, along the succession. Additionally ten squares were left in the tanks for 3 additional weeks but only the biomass pattern was determined.

### Multifractal analysis

To characterize the scaling behavior of the system we estimated the Fourier power spectrum of the biomass spatial pattern [39]. Power laws characteristics of scale invariance were observed. The exponents obtained from these can be used to determine stationarity of the dataset [40], here we refer to the broad sense definition of stationarity that means stationarity of spatial autocorrelation function.

Although fractal analysis can be used with nonstationary data [41], stationarity was a desirable property to obtain stable and accurate estimates [42], [43].

The estimated exponents were in all cases greater than one so we used a gradient to transform the biomass data and obtain a stationary data set (Figure S1) [40].

Thus, we used the following transformation :
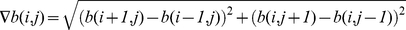
were *b*(i,j) is the original biomass data, i and j have a range determined by the resolution of the image. We normalized the data using:
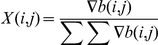



After that we divide the spatial dataset with boxes of size *ε*, and compute:
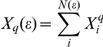
being *N*(*ε*) the number of boxes needed to cover the image and *q* a real number called moment order.

We then estimated the exponents *K*(*q*):
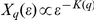
as the slope of *log(X_q_)* versus *log(ε)* using standard least squares.

A nonincreasing hierarchy known as generalized or Renyi dimensions [44], [45] can be defined *D*(*q*) = *K*(*q*)/(*q*-1). The most important exponent in this approach is quite possibly *D*(*1*), the information dimension. When *q* = 1 *D*(*q*) is undefined, so it is estimated replacing *log(X_q_)* by the following:
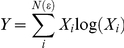
thus *D*(*1*) is the slope of *Y* versus log(*ε*).

*D*(*1*) provides us with a straightforward measure of the degree of intermittency or heterogeneity in the system [46]. Also the exponent *D(1)* characterize the distribution and intensity of singularities, i.e. of high values of biomass with respect to the mean. If *D(1)* is smaller the distribution of singularities will be more sparse and data will be more spiky, if *D(1)* is greater (approaching two in our case) the singularities will have lower values of biomass and a more uniform distribution.

Other important values of *D(q)* are for *q* = 0, *D(0)* is the dimension of the support of the distribution known as the standard fractal dimension. In our case, it is always two because the periphyton fills all the plate. For *q* = 2, *D*(*2*) is a measure of scaling of spatial correlation: the correlation dimension. But the complete spectrum provides us with an enormous amount of additional information about the geometric structure of the underlying fractal distribution [27]. For a general interpretation positive *q* preferentially weight the denser regions of the distribution while negative *q* quantify the sparse regions of it [47].

A statistical permutation test was used to compare pairs of *D(q)* curves over the course of succession. The test statistic was the two-sample *t*-statistic to compare *D(q)* values between weeks, averaged over *q*. A *P*-value was obtained for the test statistic by simulation. Plates were randomly allocated to each of two groups (different weeks) and the mean *t* was recalculated for 10000 data sets permuted in this way [48]. Pairwise comparisons were performed between the weeks. The *P*-values were adjusted for multiple testing using a step-down Bonferroni procedure [49]. The statmod R package was used to perform these tests [50].

To determine if *D(q)* spectra are different from one produced by a random spatial distribution of biomass we performed a randomization test. We shuffle the pixel position and recalculate *D(q)* and obtained a confidence interval for a random pattern performing 1000 repetitions [48]. If the actual values of *D(q)* falls outside the interval the spatial pattern is not random.

Kendall's rank correlation and quantile regression were used to relate *D(1)* with Shannon's diversity across the weeks. Quantile regression allows the examination of the maximum response, rather than the mean response, of one variable to a predictor.

It can be used for analyzing data with non-constant variance, which is often meaningful for ecological processes in which many unmeasured variables may affect the response The ecological concept of limiting factors as constraints on organisms often focuses on rates of change in quantiles near the maximum response, when only a subset of limiting factors are measured [51].

## Results

**Figure 1:**
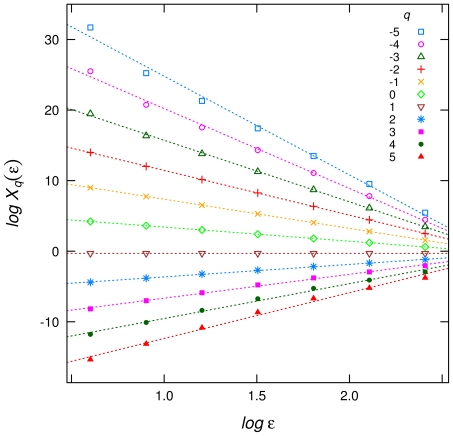
A typical graph in logarithmic scale corresponding to 3 weeks of development of the biomass spatial distributions of X*_q_* versus *ε* for *q* varying from -5 to 5, showing a very good linear fit for all q considered, the R^2^ values were always greater than 0.99. All other biomass distributions showed similar fits with R^2^ values between 0.94 and 0.99.

A data set can be called multifractal if the plots of log *X_q_*(*ε*) vs log(*ε*) are straight lines for wide range of log(*ε*) and several values of *q*. The biomass data from the photographs satisfy this assumption over the range of scales considered (Figure 1 and figures S2, S3, S4, S5, S6, S7, S8). The data used to calculate *X_q_* at each scale was not independent because the squares used for smaller *ε* values are nested in the greater ones. This violates the assumptions implicit when performing statistical tests but does not invalidate the least square method to determine the exponents *K(q)*. The coefficient of determination R^2^ can be used as a descriptive measure of goodness of fit [28]. The vast majority of R^2^ were greater than 0.99, and never were less than 0.94.

**Figure 2:**
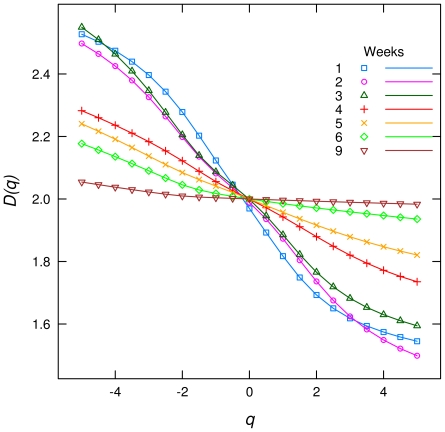
Spectrum of generalized dimensions *D(q)* for different times of the periphyton succession. The graphs are averages for all the plates with the same weeks of development.

**Figure 3:**
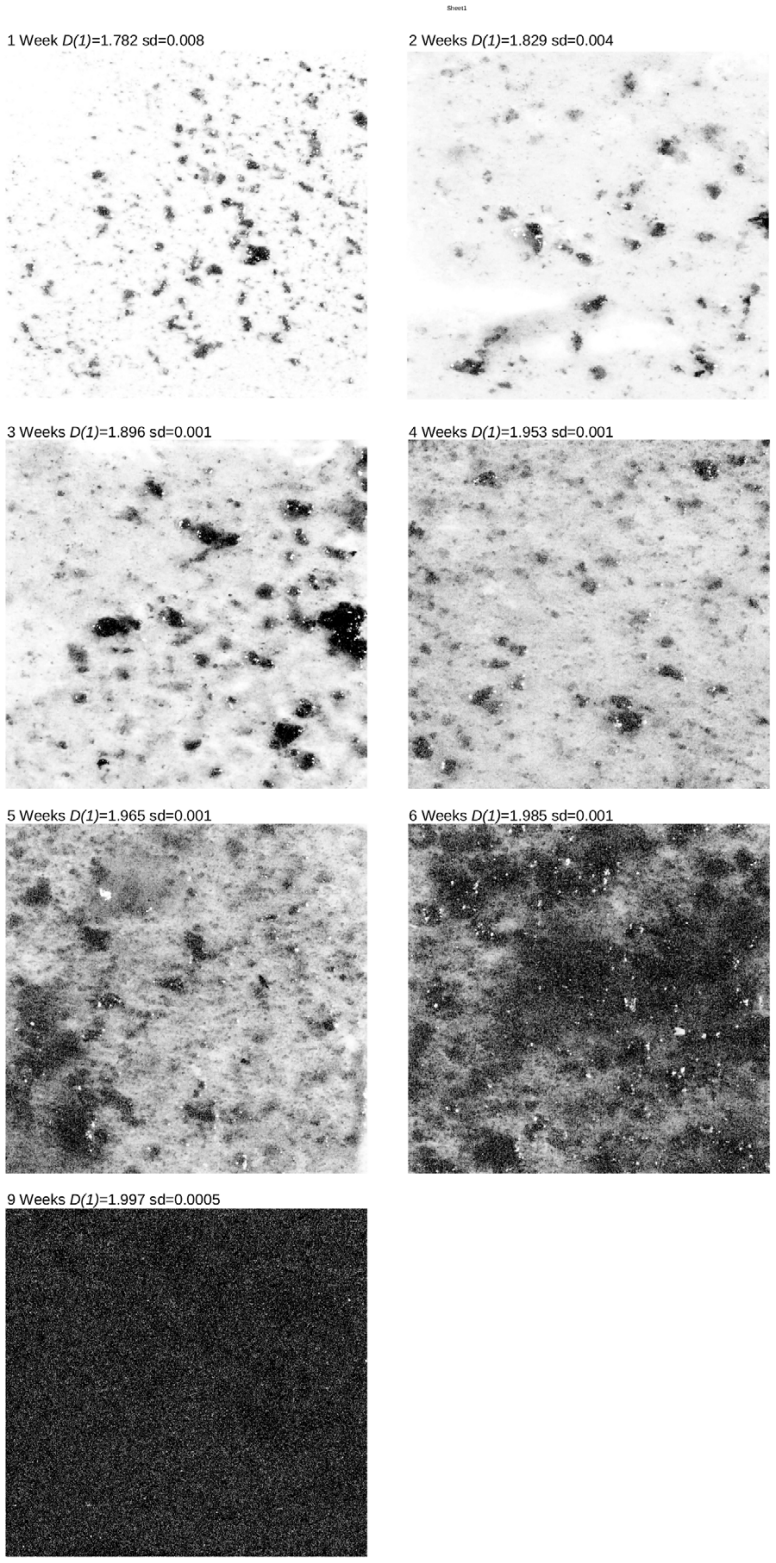
Images of periphyton biomass for different weeks of development. D(1) is the information dimension, and sd the standard deviation.

The *D(q)* vs. *q* plot, show that the “singularities” in the spatial distribution of biomass decrease with time, i.e. the curves become flatter (Figure 2). This pattern can be observed looking at the biomass images of the community development (Figure 3). All weeks have significant differences (P<0.01) except weeks one and two. Looking at the images of biomass, weeks 1 and 2 appear to be different, but analysing the standard deviation of *D(1)* (Table 1) we realize that in these weeks the s.d. is much greater than in the rest of the weeks. This means that the succession development is more variable in its first stages, in fact, the s.d. tends to dimish as the succession advance suggesting a convergence of spatial pattern by the end of succession.

**Table 1:** Information dimension *D(1)* plus standard deviation, number of species and Shannon diversity (*H*) calculated for each week of the periphyton succession.

	Weeks						
	1	2	3	4	5	6	9
*D(1)*	1.780±0.038	1.881±0.039	1.897±0.013	1.943±0.017	1.957±0.006	1.985±0.002	1.996±0.001
No.Species	20	11	19	12	16	23	–
*H*	3.57	2.69	2.26	2.11	2.77	1.62	–

Each week, the values of *D(q)* are closer to two than the values of the previous week. This monotonic relationship is maintained for all *D(q)* but the negative values of the second and third week, which are inverted. Regions of lower biomass of the third week have more irregularities than ones from the second week.

**Figure 4:**
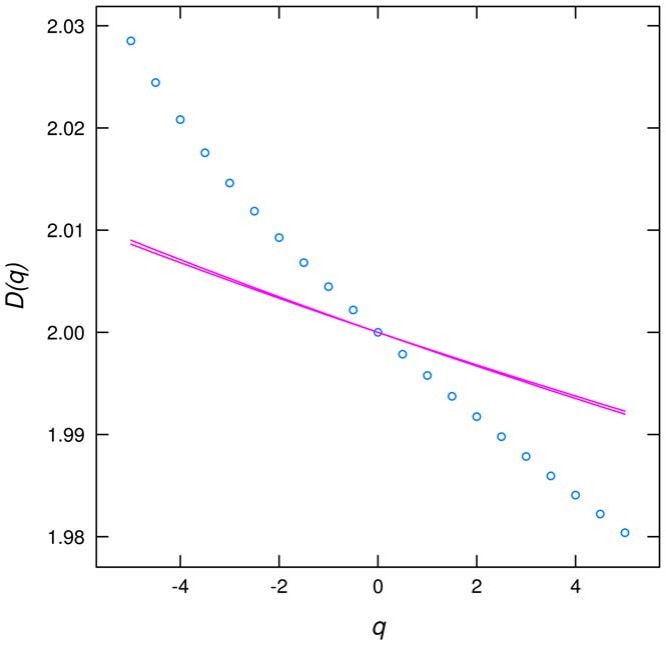
Example of confidence intervals (P = 0.01) for generalized dimensions *D(q)* for one plate of 9 weeks. These were calculated using a randomization of biomass spatial distribution and indicates that the spatial distribution is not random. All other plates give similar results.

At the 9th week *D(q)* varies very little with *q* and is very close to two. A uniform surface with equal values of biomass will give a straight line at two, so we could say that the biomass is close to not being a multifractal anymore. There is very little structure on the image of 9 weeks, the dominant feature seems to be random noise produced by the method of biomass estimation. Indeed there is some structure because the test for random spatial distribution was rejected for all q, for all plates and weeks (Figure 4). This stage is rarely reached in natural scenarios because spates in streams and other natural disturbances remove most of the attached biomass and reset succession to its first phases.

**Figure 5:**
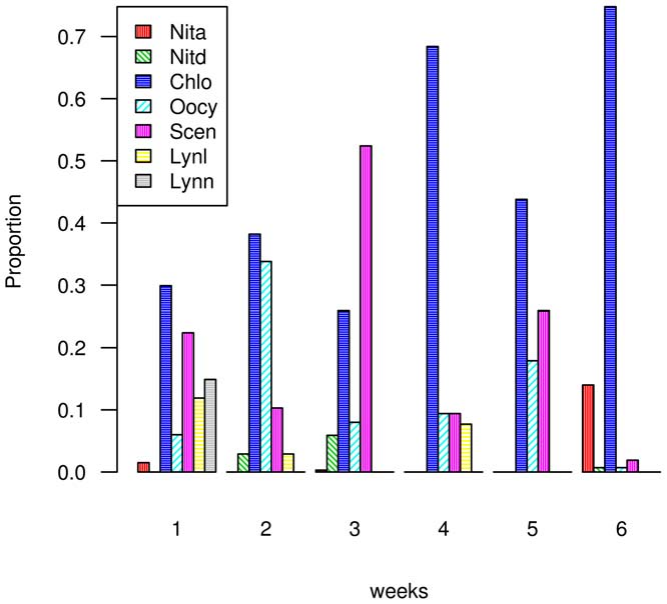
Proportion of the most abundant species for each week. The species that provide more than 5% of the total abundance for each week are included. The legend abbreviations are: Chlo: Chlorella sp, Scen: Scenedesmus sp, Oocy: Oocystis sp, Nita: Nitzschia amphibia, Nitd: Nitzschia dissipata, Lynl: Lyngbia limnetica, Lynn: Lyngbia nordgardii.

In the passage from the third to fourth week there is a big separation between curves, and this coincides with a change in species dominance from *Oocystis sp.* to *Chlorella sp.* (Figure 5). This is the the only place where this kind of change occurs and can be related to a structural change in the community.

*Chlorella sp* is the only species occurring at high densities along the whole succession. This species has a cyclical pattern of dominance. In the second week it co-dominates with *Oocystis sp*, and in the third week the community is dominated by *Scenedesmus sp*, replacing *Chlorella sp* (Figure 5). The number of species is around 20 and has a cyclic behavior with peaks at one, three and six weeks and the Shannon diversity index *H* decreases as the succession advances, except for the 4th week where *H* is higher than the previous week (Table 1). The information dimension *D(1)*, used to characterize spatial heterogeneity, increases along the succession. This means that spatial heterogeneity decreases since a *D(1)* approaching two implies a flatter surface (Table 1).

**Figure 6:**
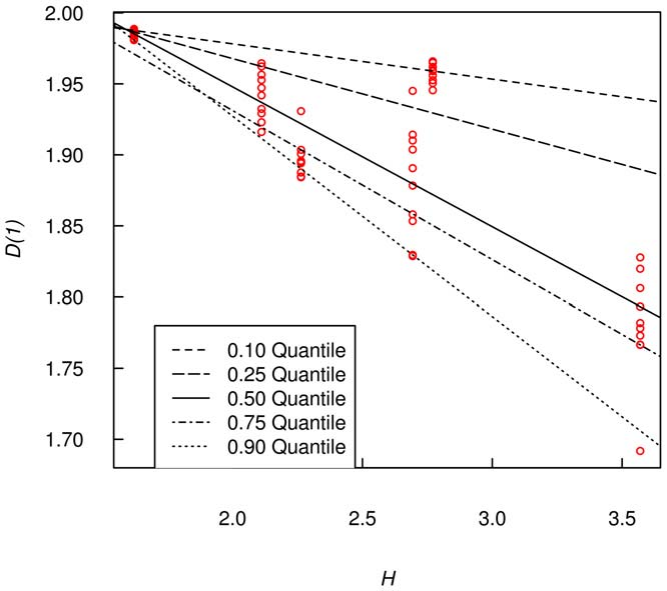
Information dimension *D(1)* by Shannon's diversity index *H*. The lines are estimated using quantile regression, all slopes are statistically different from zero ( P<0.05), and all slopes are less than zero indicating a negative relationship with diversity which means a positive relationship with heterogeneity.

The *D(1)* seems to be related to the Shannon diversity index *H*, Kendall's rank correlation is highly significant (tau = −0.52, P<0.05) (Figure 6). All quantiles are different from zero (P<0.05) and all of them show a decreasing tendency, which means that the higher H is, the lower *D(1)*, and the more heterogeneous is the biomass spatial distribution. The 90% quantile can be used as a guide of the minimum diversity of a site given its *D(1)* (Figure 6).

In fact *H* and *D(1)* are both expressions of Shannon entropy, one estimated from the biomass distribution and another from species abundances [52]. Thus we found a way to relate the information contained in the spatial pattern to the information contained in the abundance and the number of species in this ecosystem.

## Discussion

Abiotic systems maintained far from equilibrium can produce macroscopic ordered patterns in space emerging from what looks like microscopic chaos. This is especially evident when dealing with biotic ecosystems where similar patterns appear replicated at different scales [27], [53].

Starting from homogeneous substrata the colonizing microalgae produces a self-similar spatial pattern. Thus the spatial pattern is not imposed by external conditions but is generated endogenously by the interactions and growth of algae and other components of the periphyton, therefore the conditions for self-organization are met [54]. This is the first part of our hypothesis: the spatial pattern of periphyton communities is self-organized.

The system produces a self-similar spatial pattern resembling those occurring in critical states, a description of the kind of criticality and the possible underlying mechanisms is outside of the scope of this paper and is reviewed elsewhere [55].

The model of cycles of heterogeneity assumes that when one species replaces another a high heterogeneity should be observed. We noted that in the third week of development, the two codominant species (*Chlorella sp* and *Oocystis sp*) were replaced by *Scenedesmus sp*, but we did not observe an increase in heterogeneity as measured by the information dimension *D(1)*. In fact we see that *D(1)* always decreases over time, but this could occur because the sampling interval is one week and the replacement of species has already been completed. A greater heterogeneity was only detected for negative *q*, that means that the lower biomass regions the plates are more heterogeneous. This evidence, which can only be observed using the multifractal approach, could lend partial support to the model of cycles of heterogeneity.

The link between persistence and fractal dimension has been studied in the context of patch dynamics. Both theory and experimentation have shown that when patch limits are more intricate and complex, these correspond to early succession stages, with less persistent patches and higher fractal dimension [13], [56]. On the other hand when patches are more uniform, fractal dimension is lower corresponding to late successional stages. In our case *D(1)* has a different meaning: when it is lower it corresponds to more complex and heterogeneous spatial biomass distributions. When it is higher, closer to two, it corresponds to more uniform distributions. In fact when *D(1)* is equal to two the distribution is a completely flat surface. Our observations confirm previous studies, in that early successional stages correspond to more complex distributions and late successional to more uniform ones. This could be useful for applications in landscape management and classification of different areas by remote sensing [12]. The *D(q)* spectra can be used to characterize different successional stages, but more studies are needed to see if it can be used in systems with habitat heterogeneity.

The patch distribution of vegetation was extensively studied in arid regions [57]. It has been hypothesized that the loss of a self-organized spatial pattern in the vegetation patchiness can be used as signature of imminent catastrophic shifts between alternative states [54]. Evidence that patch distributions are self-similar (fractal) was found in Mediterranean ecosystems and it was also found that with increasing grazing pressure, vegetation patterns deviated from self-similarity [58]. Thus the loss of self-similar behavior can be used as a warning signal for the onset of desertification. In our case the loss of self-similiarity can be related to the loss of biodiversity. Which is not a catastrophic change but a gradual change produced by succession.

We observed a relationship between the information dimension estimated from the spatial pattern, that is from a snapshot of the community, and the Shannon's diversity estimated from the species abundances. To put it in another way, we could infer a minimum diversity of a site without either species identification nor estimation of abundances. As the spatial distribution of periphyton's biomass tends to lose the fractal behavior, diversity also tends to decrease. This is observed when the information dimension approaches an integer value of two.

The negative relationship between biodiversity and *D(1)* was also found in semi-arid shrubland and grasslands ecosystems [59], [60]. In that case the relationship can be produced by an increased habitat heterogeneity that permits the coexistence of a greater number of species, or by the self-organization process, but is difficult to separate the two possible sources of heterogeneity. In our case we are confident that the spatial heterogeneity is produced by self-organization, i. e. by the interaction of species in the succession process.

This relationship may be due to different algal growth forms and thus estimating functional diversity could provide a more exact picture. Other studies have found a positive relationship between functional diversity and spatial biomass stability [6], which means less heterogeneity. This is opposite to our results, but the heterogeneity was measured at only one scale and the spatial extent used in that study was very limited compared to ours, so their results may not be comparable to ours.

Our approach could be applied to other ecosystems where the biomass distribution can be estimated, such as tropical forest [61], and used to identify zones with high and low diversity as a warning signal for conservation planning and management.

We are in the process of building a model that can reproduce the self-similar patterns and the link between Shannon's diversity and the information dimension. More detailed studies and the extension to other ecosystems are essential to evaluate the relevance and applicability of the results presented here.

## Supporting Information

Figure S1 Bidimentional power spectrum for the periphyton's biomass distribution
(PDF)

Figure S2 Graph in logarithmic scale of *X_q_* versus *ε* for 1 week of community development
(PDF)

Figure S3 Graph in logarithmic scale of *X_q_* versus *ε* for 2 weeks of community development
(PDF)

Figure S4 Graph in logarithmic scale of *X_q_* versus *ε* for 3 weeks of community development
(PDF)

Figure S5 Graph in logarithmic scale of *X_q_* versus *ε* for 4 weeks of community development
(PDF)

Figure S6 Graph in logarithmic scale of *X_q_* versus *ε* for 5 weeks of community development
(PDF)

Figure S7 Graph in logarithmic scale of *X_q_* versus *ε* for 6 weeks of community development
(PDF)

Figure S8 Graph in logarithmic scale of *X_q_* versus *ε* for 9 weeks of community development
(PDF)
