# Supplementary material for: Multifractal Spatial Patterns and Diversity in an Ecological Succession
Source: PLoS One. 2012 Mar 21;7(3):e34096. doi: 10.1371/journal.pone.0034096 (PMC3312349; doi:10.1371/journal.pone.0034096)
Supplement: Figure S1 — Bidimentional power spectrum for the periphyton's biomass distribution (PDF) [file pone.0034096.s001.pdf]

Figure S1. Log-log plot of the Bidimentional power spectrum for the periphyton's biomass distribution. Where E(k) is the value of the spectrum often called energy spectrum an k is the frequency. The values of E(k) are averaged for each week. Beta is the slope of the lines estimated over a fixed range for all weeks, all slopes are greater than one, indicating non-stationarity in the data this justifies the application of a gradient to estimate the multifractal spectrum (Davis et al. 1996).

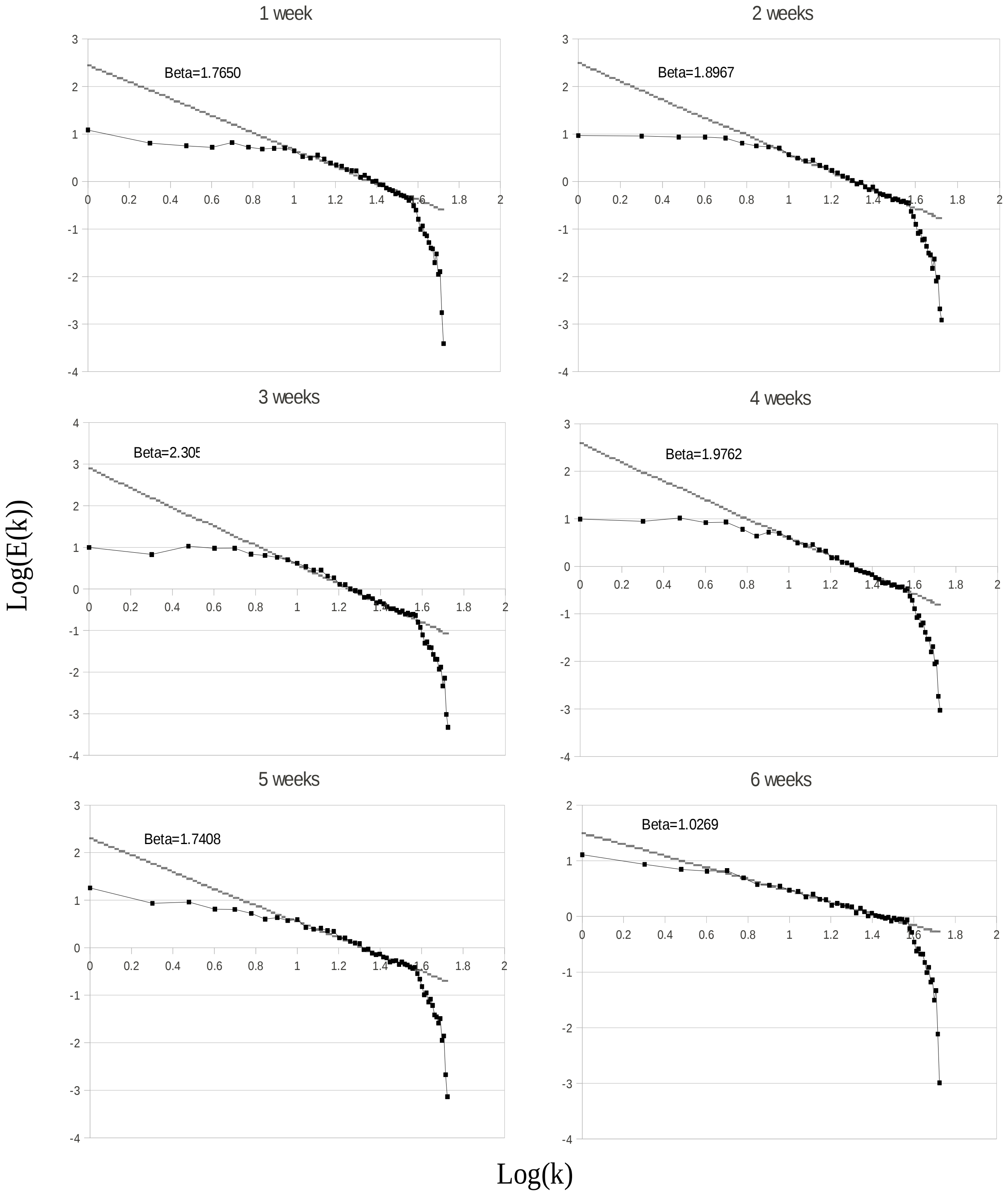

Bibliography

Davis, A., Marshak, A., Wiscombe, W. & Cahalan, R. (1996). Scale invariance of liquid water distributions in marine stratocumulus. Part I: Spectral properties and stationarity issues. *Journal of Atmospheric Sciences*, 53, 1538-1558.
