# Supplementary material for: Multifractal Spatial Patterns and Diversity in an Ecological Succession
Source: PLoS One. 2012 Mar 21;7(3):e34096. doi: 10.1371/journal.pone.0034096 (PMC3312349; doi:10.1371/journal.pone.0034096)

Figure S3. A graph in logarithmic scale of X_q versus ε for q varying from -5 to 5, showing a linear fit for all q considered, corresponding to the plates with 2 weeks of community development. The R^2 values were between 0.986 and 0.999. The label number refers to the original image number.

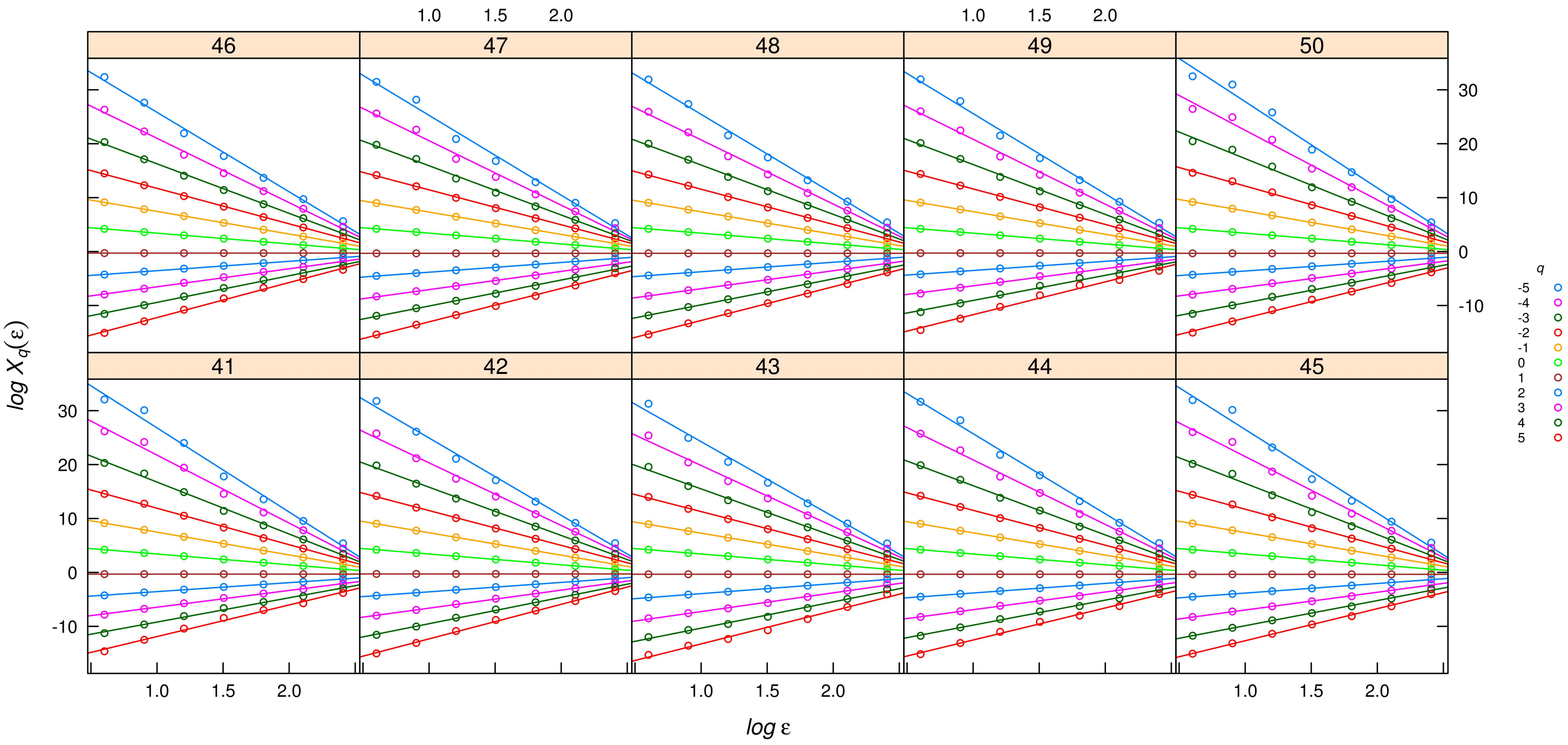

Supplement: Figure S3 — Graph in logarithmic scale of Xq versus ε for 2 weeks of community development (PDF) [file pone.0034096.s003.pdf]
